# Connectivity in gene coexpression networks negatively correlates with rates of molecular evolution in flowering plants

**DOI:** 10.1371/journal.pone.0182289

**Published:** 2017-07-31

**Authors:** Rishi R. Masalia, Adam J. Bewick, John M. Burke

**Affiliations:** 1 Department of Plant Biology, University of Georgia, Athens, Georgia, United States of America; 2 Department of Genetics, University of Georgia, Athens, Georgia, United States of America; University of Toronto, CANADA

## Abstract

Gene coexpression networks are a useful tool for summarizing transcriptomic data and providing insight into patterns of gene regulation in a variety of species. Though there has been considerable interest in studying the evolution of network topology across species, less attention has been paid to the relationship between network position and patterns of molecular evolution. Here, we generated coexpression networks from publicly available expression data for seven flowering plant taxa (*Arabidopsis thaliana*, *Glycine max*, *Oryza sativa*, *Populus* spp., *Solanum lycopersicum*, *Vitis* spp., and *Zea mays*) to investigate the relationship between network position and rates of molecular evolution. We found a significant negative correlation between network connectivity and rates of molecular evolution, with more highly connected (i.e., “hub”) genes having significantly lower nonsynonymous substitution rates and *dN*/*dS* ratios compared to less highly connected (i.e., “peripheral”) genes across the taxa surveyed. These findings suggest that more centrally located hub genes are, on average, subject to higher levels of evolutionary constraint than are genes located on the periphery of gene coexpression networks. The consistency of this result across disparate taxa suggests that it holds for flowering plants in general, as opposed to being a species-specific phenomenon.

## Introduction

**Fig 1:**
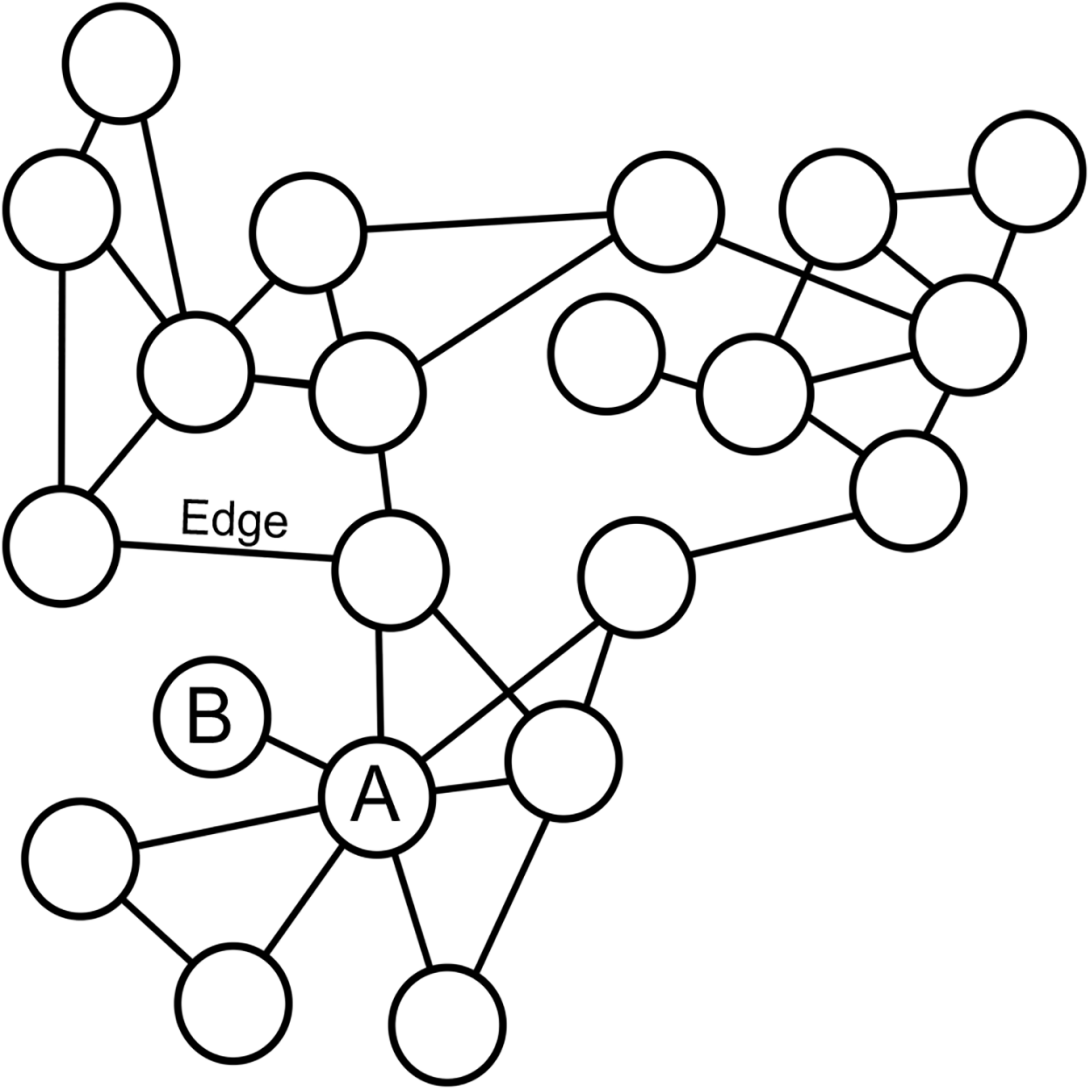
Simplified representation of a hypothetical coexpression network. Node A represents a hub gene while node B represents a peripheral gene. Lines connecting nodes represent network edges, and reflect correlations in expression.

In recent years, transcriptomic analyses have become a standard tool in many laboratories (e.g., [1–3]). Driven by the availability of high-density arrays and the ongoing improvement of nucleotide sequencing platforms, massive amounts of transcriptomic data have thus been produced (e.g., [3,4]). While much attention has been paid to patterns of differential expression and instances of alternative splicing across experimental perturbations, many have sought to place transcriptomic data into a broader biological framework via the construction of gene coexpression networks (e.g., [5–11]). Coexpression networks are composed of a series of nodes (i.e., genes) and edges (i.e., connections) that reflect correlations in gene expression (Fig 1). These networks are often constructed from multiple tissue types, developmental stages, and/or experimental treatments, providing a holistic view of gene coexpression.

Once a coexpression network has been generated, it can be subdivided into ‘modules,’ which are suites of highly interconnected, or coexpressed genes. Within modules, new candidate genes for a trait of interest can be identified based on associations with genes previously linked to these traits, an approach known as ‘guilt-by-association’ [12–14]. Such analyses have thus emerged as a common approach for functional prediction (e.g., [15–18]), and have been performed in a wide variety of species (e.g., *Arabidopsis thaliana*: [14,19–23]; *Homo sapiens*: [24]; *Mus musculus*: [25]; *Oryza sativa*: [9,26,27]; and *Zea mays*: [27]). There has also been substantial interest in the evolution of network topologies across taxonomic groups and over time (e.g., [28–32]). Less attention has, however, been paid to the influence of network topology on the evolution of genes contained within such networks.

Interestingly, analyses of protein-protein interaction networks have revealed that the deletion of more centrally located genes is often lethal and that connectivity in such networks is negatively correlated with rates of protein evolution (e.g., [33–40]). These findings suggest that more centrally located proteins, which have more direct molecular interactions (i.e., connections), are more likely to be essential and/or subject to stronger purifying selection than those located on the periphery [6,41]. This pattern may reflect the involvement of such genes in more biological processes, thereby placing them under greater pleiotropic constraint, though the biological significance of this correlation has been debated [42]. Similarly, the position of a gene within a biochemical pathway has been found to influence rates of molecular evolution, with genes found earlier in pathways exhibiting evidence of greater functional constraint than their downstream counterparts (e.g., [43–48]. Once again, this pattern may be due to differences in pleiotropic constraint, with mutations in genes acting earlier in a pathway having more potential downstream consequences than those in genes later in the pathway. It has been suggested that a similar relationship should also hold in coexpression networks [6], with more centrally located genes experiencing greater selective constraint. Two recent studies in plants [49,50] have supported this prediction, though the extent to which this pattern holds across plant species in general remains an open question.

Here, we investigate the relationship between the position of a gene in a coexpression network and its rate of molecular evolution in seven disparate angiosperms. We hypothesize that more centrally located (i.e., hub) genes (e.g., gene A in Fig 1) will, on average, experience greater levels of functional constraint due to their potential involvement in a larger number of biological processes as compared to genes located on the periphery. As such, these genes should exhibit lower nonsynonymous substitution rates as compared to more peripheral genes (e.g., gene B in Fig 1). To test this hypothesis, we leveraged existing datasets to analyze genome-wide patterns of coexpression in *Arabidopsis thaliana*, *Glycine max*, *Oryza sativa*, *Populus* spp., *Solanum lycopersicum*, *Vitis spp*., and *Zea mays* and used the resulting networks to examine patterns of molecular evolution as a function of connectivity.

## Materials and methods

Raw microarray datasets were downloaded from the Gene Expression Omnibus (GEO), ArrayExpress, and/or The Arabidopsis Information Resource (TAIR, for *A*. *thaliana* only) corresponding to 2501, 1163, 1572, 1020, 385, 517, and 627 experiments for *A*. *thaliana*, *G*. *max*, *O*. *sativa*, *Populus* spp., *S*. *lycopersicum*, *Vitis* spp., and *Z*. *mays*, respectively. The poplar and grape arrays were utilized at the genus level, and represent 13 and 4 species, respectively. As is common with coexpression networks, these arrays sample a large breadth of experimental data spanning multiple tissues types, developmental stages, and stress treatments (both abiotic and biotic). A complete list of selected experiments and arrays, including their GEO or ArrayExpress IDs along with a brief description, is provided in S1 Appendix. Expression intensities were extracted from all selected microarray experiments and normalized for each taxon using the Robust Multichip Average (RMA) method implemented through the Bioconductor package Affy in R [51]. After data normalization, unique probe-to-gene matches were identified, where the gene corresponds to the GenBank or Phytozome unique identifier provided in the array. If multiple array probes matched a given gene, one of these probes was selected at random to eliminate duplicate data. Genes with variable expression were identified by assessing the distribution of the coefficient of variation (CV) of expression, and only those genes with a CV found within the upper 95% confidence interval of CV were retained. A complete list of retained genes and probes can be found in S2 Appendix.

To determine gene connectivity, the R package Weighted Gene Co-expression Network Analysis (WGCNA) [52] was used to construct coexpression networks for all seven plant taxa. Briefly, WGCNA calculates a Pearson’s correlation matrix for all genes (95% confidence interval), and transforms this matrix by raising all values to a power β (soft thresholding). The β value for a given taxon is a nonlinear transformation, which can influence the correlation between any two genes [53], weighting those with higher connectivity over those with lower. This influences the shape of network modules and creates a scale-free topology. We estimated a β value for each taxon based on underlying expression values using the function *pickSoftThreshold* in the WGCNA package, resulting in power values of 2, 5, 13, 3, 9, 5, and 4 for *A*. *thaliana*, *G*. *max*, *O*. *sativa*, *Populus* spp., *S*. *lycopersicum*, *Vitis* spp., and *Z*. *mays*, respectively (S1 Fig). All remaining parameters were kept at the recommended default values as stated in the manual. Estimates of per gene connectivity were extracted from the output by tallying the number of genes connected to each unique gene in a network (S2 Appendix). Network characteristics for all seven taxa are summarized in S3 Appendix.

Once connectivity was established, pairwise rates of molecular evolution were estimated for each taxon using PAML’s yn00 model [54]. For comparing relative rates of molecular evolution between taxa, a monocot (*O*. *sativa*) or dicot (*A*. *thaliana*) outgroup was used for the dicot or monocot species, respectively. For all seven taxa, sequence CDS files were gathered or generated to match with array probes. For *A*. *thaliana*, *O*. *sativa*, and *G*. *max*, these CDS files were downloaded from Phytozome v10.1 (http://phytozome.jgi.doe.gov/pz/portal.html), while sequences for the other species were downloaded from GenBank by matching probe annotations to GenBank sequence accession numbers. Putative orthologs between each taxon and its outgroup were estimated using reciprocal best BLAST hits with an e-value threshold of <1E-08. Sequence pairs were aligned using MUSCLE v.3.8 [55], and in-frame stretches of aligned sequence (≥ 30 bp) were identified and concatenated into a single contiguous sequence of ≥ 300 bp prior to analyses in PAML, using custom perl scripts (mani-seq; https://github.com/bewickaj). All orthologous sequence pairs can be found in S2 Appendix. An estimate of per gene connectivity was determined for each taxon (range of all taxon-wide averages: 228 to 4682) using the coexpression networks generated.

**Fig 2:**
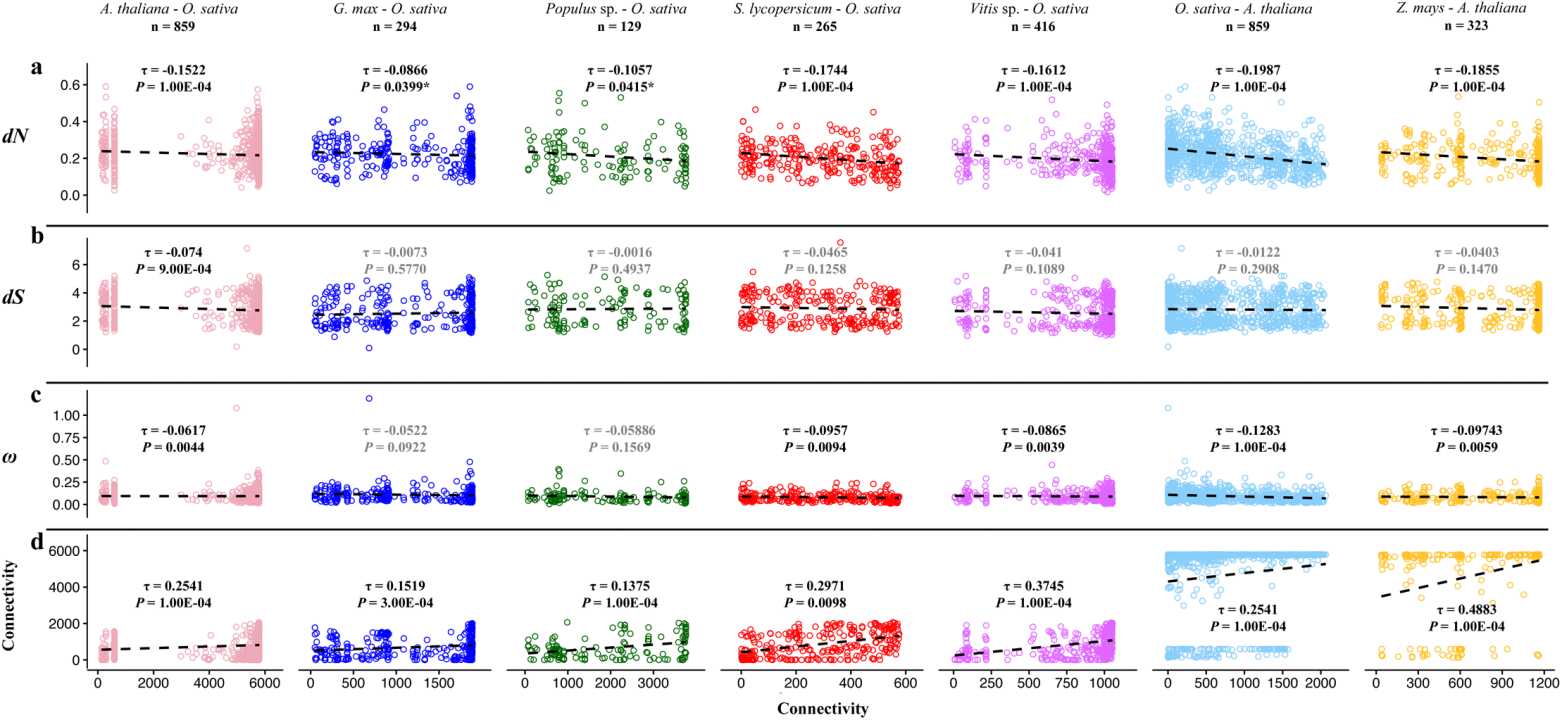
Linear regression of gene connectivity of seven taxa analyzed. Taxa: *A*. *thaliana*, *G*. *max*, *Populus spp*., *S*. *lycopersicum*, *Vitis spp*., *O*. *sativa*, and *Z*. *mays*, against (a): non-synonymous substitutions (*dN*), (b): synonymous substitutions (*dS*), (c): estimates of adaptive evolution (ω = *dN*/*dS*) and (d): number of connections in ortholog comparison. Circles represent genes, while the regression coefficient, represented as Kendall's tau (τ) coefficient, is the dashed line. Significance is indicated by bold text. Note that all significant results except the two marked with an asterisk (*) remained significant after correcting for multiple comparisons (see text for details).

Rates of nonsynonymous substitutions per nonsynonymous site (*dN*), synonymous substitutions per synonymous site (*dS*), and estimates of adaptive evolution (*ω = dN/dS*) were visualized via linear regression against our estimates of gene connectivity (Fig 2). Significance of the correlations, estimated as Kendall's tau (*τ*) to address tied correlation ranks, was assessed via randomization tests. Briefly, this involved randomizing the parameter of interest vs. connectivity for each comparison in Fig 2 and recalculating the correlation. This procedure was repeated 10,000 times to generate a null distribution against which the observed values were compared. To account for multiple comparisons across taxa, we applied a sequential Bonferroni correction at α = 0.05 [56, 57]. All statistical tests were performed in R [58].

## Results

In terms of the relationship between connectedness and evolutionary constraint, our results align with what has previously been found in biochemical pathways and protein-protein interaction networks. Our analyses (based on n = 859, 294, 139, 265, 416, 859, 323 orthologous sequence pairs for *A*. *thaliana*—*O*.*sativa*, *G*. *max*—*O*. *sativa*, *Populus* spp—*O*. *sativa*, *S*. *lycopersicum*—*O*. *sativa*, *Vitis* spp.—*O*. *sativa*, *O*. *sativa*—*A*. *thaliana*, *Z*. *mays*—*A*. *thaliana*, respectively) revealed that the nonsynonymous substitution rate (*dN*) was significantly negatively correlated with connectivity in the majority of taxa investigated (the results for *G*. *max* and *Populus* spp. were nominally significant based on our randomization tests, but not significant after controlling for multiple comparisons; Fig 2A). The same overall pattern was evident for our estimates of adaptive evolution (*ω = dN/dS*) (Fig 2C), though the correlations between *ω* and connectedness were generally weaker than those between *dN* and connectedness. While the correlations between *dN* or *ω* and connectedness were not significant for two of the seven taxa (*G*. *max* and *Populus* spp.), they did exhibit the same overall trend (i.e., a negative relationship) as was observed in the other five taxa. When combining our results across all seven taxa, we found that the overall pattern–i.e., more highly interconnected hub genes exhibited stronger evolutionary constraint–was highly significant (Fisher’s combined probability test, *P* < 1E-08 for both *dN* and *ω* [using CombinePValue; https://CRAN.R-project.org/package=CombinePValue]; S2 Appendix). The synonymous substitution rate (*dS)* was only significantly correlated with connectivity in the *A*. *thaliana*—*O*. *sativa* comparison (Fig 2B). While all *dS*-related correlations had the same (negative) sign, resulting in a combined probability of *P* < 1E-02, it is noteworthy that this result is largely attributable to the *A*. *thaliana*—*O*. *sativa* comparison (*P* = 0.0009, with the other six *P*-values ranging from 0.11–0.58). Despite overall differences in the numbers of connections amongst genes within a species, the connectivity of orthologous gene pairs between each taxon and its outgroup (i.e., *A*. *thaliana* for monocots and *O*. *sativa* for dicots) was positively correlated (all *P* < 0.05, Fig 2D; S4 Appendix).

## Discussion

Taken together, these findings indicate that more centrally located and highly interconnected (i.e., hub) genes exhibit reduced nonsynonymous substitution rates and rates of adaptive evolution. This observation is consistent with our hypothesis that such genes are subject to greater functional constraint than less connected genes that can be found on the periphery of coexpression networks. The consistency of this result across disparate taxa suggests that it holds for flowering plants in general, as opposed to being a species-specific phenomenon. Though our results align with the findings of previous work done in both protein-protein interaction networks [33–36] and biochemical pathways (e.g., [43–47]), it is important to note that patterns of coexpression do not necessarily translate into direct molecular interactions or biochemical relationships. So why do we see this negative correlation between connectivity and rates of molecular evolution, both here and in other recent studies in plants (i.e., [49,50])? While coexpression patterns are not necessarily indicative of direct molecular interactions, it seems likely that more centrally located (i.e., hub) genes will tend to influence more aspects of organismal biology than peripheral genes, and might thus be expected to experience more antagonistic pleiotropy. That is, alterations of the amino acid sequence of a hub gene could have a multitude of associated effects, some of which may be deleterious, whereas tinkering at the periphery of a network might result in variants with fewer negative consequences.

While two recent studies have documented a significant, negative correlation between *dS* and connectivity in plants [49,50], we saw little evidence of such a correlation in our study. Indeed, only one of the comparisons (*A*. *thaliana—O*. *sativa*) revealed a highly significant (negative) correlation between *dS* and connectivity. While the remaining six comparisons resulted in negative *τ* values, none of them individually approached significance. Nonetheless, the combined probability of these results was significant (*P* < 1E-02), and the likelihood of observing seven negative correlations by chance is extremely low in the absence of a true relationship between *dS* and connectivity (two-tailed sign test: *P* < 0.05). Such a relationship could be a byproduct of mutation rate variation, with more highly connected genes experiencing fewer mutations, or it could be due to variation in selective constraint on synonymous sites (e.g., due to codon usage bias; [49]) as a function of connectedness. As noted by Josephs et al. (2017), heterogeneity in *dS* due to variation in synonymous constraint could explain our observation of a weaker correlation between ω and connectedness as compared to *dN* and connectedness.

Interestingly, a general tendency toward reduced expression variation in hub genes, evidenced in part by a paucity of “local” eQTLs associated with such genes, has also been observed [49,50]. This observation suggests that expression level variation may be subject to stabilizing selection in more highly connected genes as compared to those with fewer connections. As such, it may be that constraint on both sequence and expression changes is relaxed for genes on the periphery of coexpression networks as compared to more centrally located genes. Unfortunately, the data analyzed herein do not allow us to perform equivalent analyses, and so a more complete understanding of the relationship between expression variation and network position across flowering plants awaits further study. Likewise, a more holistic understanding of the influence of network topology on patterns of both molecular evolution and expression variation across the entirety of the plant kingdom awaits a more complete taxonomic sampling.

## Supporting information

S1 Appendix Array information on sampled taxa. (XLS)

S2 Appendix Estimated connectivity and molecular evolution estimates for retained genes and probes. (XLS)

S3 Appendix Coexpression network summary statistics per taxon. (XLS)

S4 Appendix Connectivity across orthologous pairs. (XLS)

S1 Fig Summary of soft-thresholding results across the seven taxa. (PDF)
