# Supplementary figures and images for: Connectivity in gene coexpression networks negatively correlates with rates of molecular evolution in flowering plants
Source: PLoS One. 2017 Jul 31;12(7):e0182289. doi: 10.1371/journal.pone.0182289 (PMC5536297; doi:10.1371/journal.pone.0182289)

Scale Free Topology Model Fit, signed  $R^2$

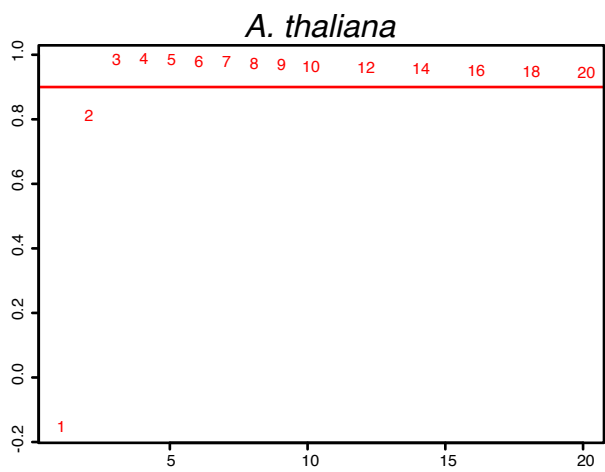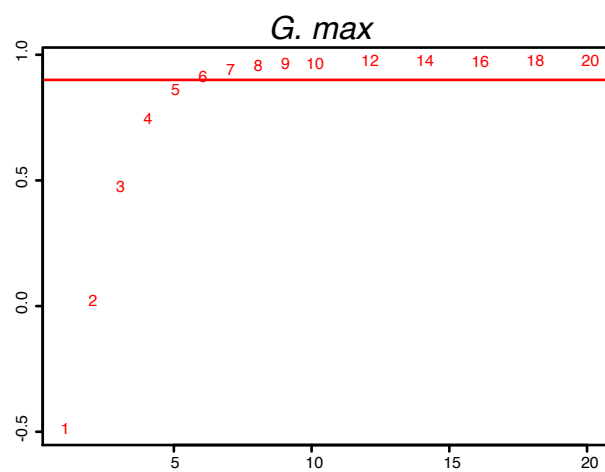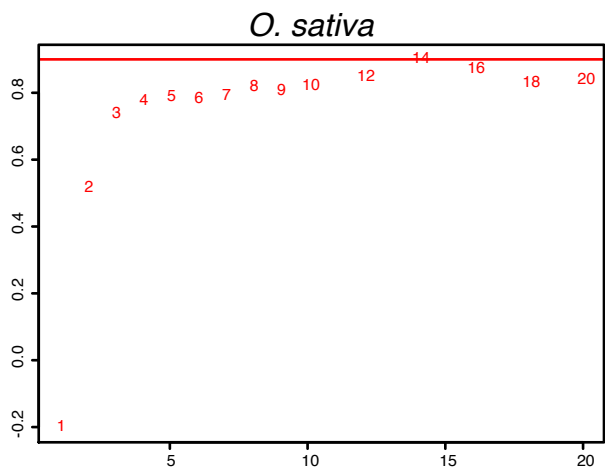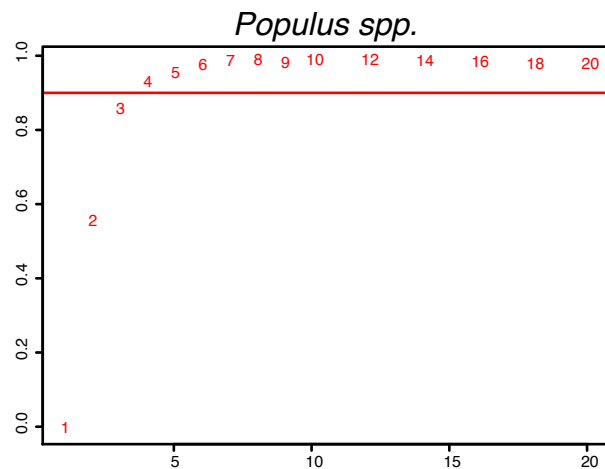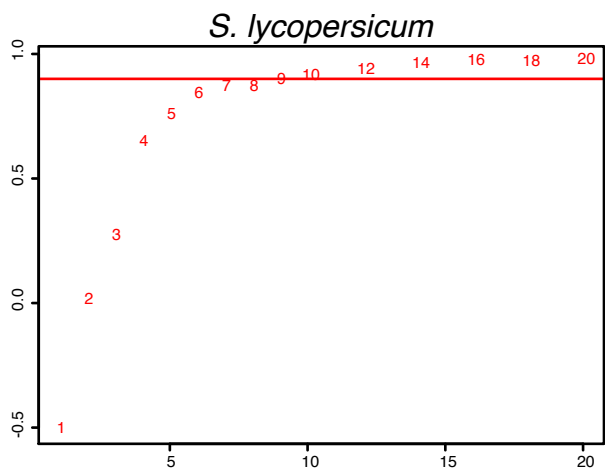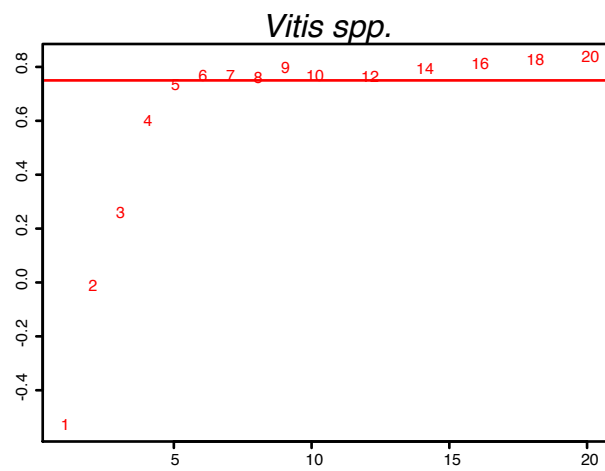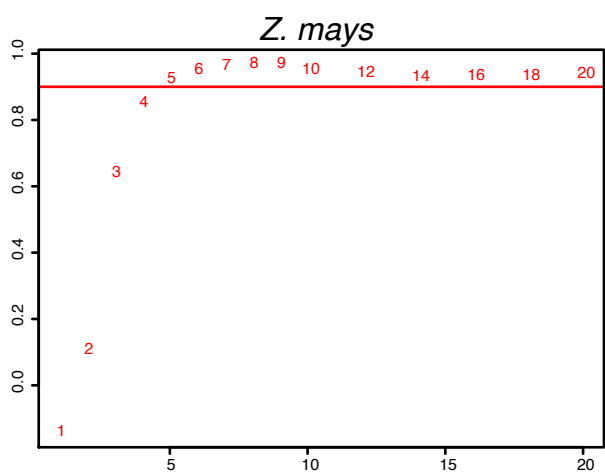

Soft Threshold (power)

Supplement: S1 Fig — (PDF) [file pone.0182289.s005.pdf]
